# Lymphovascular space involvement as a prognostic factor for survival outcomes in vulvar cancer: A systematic review and meta‐analysis

**DOI:** 10.1002/ijgo.71157

**Published:** 2026-06-29

**Authors:** Vasilios Pergialiotis, Maria Fanaki, Michail Panagiotopoulos, Vasilios Lygizos, Dimitrios Efthimios Vlachos, Nikolaos Thomakos, Dimitrios Haidopoulos

**Affiliations:** ^1^ Department of Obstetrics and Gynecology, Division of Gynecologic Oncology, “Alexandra” General Hospital National and Kapodistrian University of Athens Athens Greece

**Keywords:** LVSI, lymphovascular space involvement, meta‐analysis, survival, vulvar cancer

## Abstract

**Background:**

Lymphovascular space involvement (LVSI) is a significant prognostic factor of survival outcomes in gynecologic oncology. Synthetic studies on vulvar cancer are lacking as well as clear consensus about the importance of LSVI in vulvar cancer.

**Objectives:**

This study systematically evaluates the evidence of the impact of LVSI on vulvar cancer survival outcomes and explore its potential association with inguinofemoral metastases whenever relevant data were available.

**Method:**

We searched the international literature using the Medline, Scopus, Clinicaltrials.gov, Cochrane Central Register of Controlled Trials CENTRAL, and Google Scholar. Observational studies that compared survival outcomes among vulvar cancer patients that had positive pathology findings for LVSI and those without LVSI were included.

**Results:**

Overall, 22 articles were retrieved and six were selected for inclusion. Significant differences were noted in the progression‐free survinal (PFS) of patients with LVSI compared to that of patients without LVSI (hazard ratio [HR] 2.79, 95% confidence interval [CI] 1.27, 6.14). Similarly, the overall survival (OS) of patients with LVSI was substantially lower compared to that of patients without LVSI (HR 1.66, 95% CI 1.18, 2.35). None of the studies reported differences in lymphatic metastases among patients with LVSI and those without LVSI in the primary tumor site.

**Conclusion:**

Despite the significant heterogeneity of included studies, current data suggest that vulvar cancer patients with LVSI of the primary tumor site have worse survival outcomes. It remains unclear how much this observation is influenced by other significant factors, including the stage of the disease and positive tumor margins.

**Abbreviations**
CI: confidence intervals
ESGO: European Society of Gynecologic Oncology
HR: hazards ratio
LVSI: lymphovascular space involvement
OS: overall survival
PFS: progression free survival
PNI: prognostic nutritional index
PRISMA: Preferred Reporting Items for Systematic Reviews and Meta‐Analyses
PROSPERO: International Prospective Register of Systematic Reviews
RCT: randomized controlled trial
REM: random effects model

## 1 INTRODUCTION

Vulvar cancer primarily affects elderly women, with approximately 16 000 cases in Europe in 2020 and ranking the 19th most common cause of cancer in the female European population.^1^ Vulvar squamous cell carcinoma represents a biologically heterogeneous disease. Human papillomavirus (HPV)‐associated tumors are more common in younger women, whereas HPV‐independent tumors usually occur in older patients and might differ in clinical behavior and prognosis. According to the American Cancer Society, the 5‐year survival rate of vulvar cancer is approximately 71%.^2^ The reported incidence of recurrence in vulvar cancers ranges between 20 and 25% according to multi‐institutional studies, and if early stages are considered, this percentage only drops to 10–15%.^3^, ^4^

Stage remains the most crucial aspect of determining the need for adjuvant therapy, as patients with early‐stage disease that does not involve the surgical margins do not require further treatment.^5^ Apart from stage and positive tumor margins, several other prognostic factors have been identified as potential predictors of survival, including tumor size,^3^ perineural invasion,^6^ and lymphovascular space involvement (LVSI).^7^ However, the understanding of the impact of many of these variables remains inconsistent across studies.^8^ Therefore, clinical implications for decision making in early stage disease remain vague and limited to level IV evidence.^9^, ^10^

Lymphovascular space involvement, which refers to the presence of cancer cells within the lymphatic and blood vessels surrounding the tumor is considered a significant prognostic factor of survival in many other malignancies and directly affects the clinical decision concerning the need for adjuvant therapy.^11^, ^12^ In vulvar cancer, studies indicate that LVSI is thought to indicate a greater risk of metastasis and a more aggressive tumor behavior.^13^, ^14^, ^15^ However, to date, the European Society of Gynecologic Oncology (ESGO) has not included LVSI as a factor that should be considered as a prognostic variable of survival which could affect the decision for adjuvant therapy, with the exception of patients with surgical margins that are close to the tumor,^9^ whereas the British Gynecological Cancer Society guidelines on vulvar cancer do not even mention this variable.^5^

Considering the lack of agreement considering the actual importance of LVSI, along with evidence from various studies that seem to indicate it as a histopathological index that is negatively associated with patients' survival, we conducted a systematic review and meta‐analysis to evaluate the quality of the evidence, the gaps in current literature and the cumulative effect of LVSI on survival outcomes of vulvar cancer patients. Actual impact of this histopathological variable.

## 2 METHODS

### 2.1 Protocol registration

The Preferred Reporting Items for Systematic Reviews and Meta‐Analyses (PRISMA) criteria were followed in conducting the systematic review, which was pre‐registered in PROSPERO (International Prospective Register of Systematic Reviews) with registration number CRD42024545939.^16^ Since all of the data came from research that was published in global literature, institutional review board permission and patient consent were not required.

### 2.2 Eligibility criteria, information sources, search strategy

Studies were included based on predefined qualifying requirements. If the outcomes of interest were present, all studies that looked at how LVSI affected the survival outcomes of patients with vulvar cancer were potentially eligible for inclusion. Although the main goal of the study was to assess how LVSI affected the survival outcomes of patients with vulva squamous carcinoma, all histology groups were deemed eligible for inclusion during study retrieval, and if survival data from multiple histology groups were available, subgroup analysis was chosen. Although all vulvar cancer histological subtypes were considered during study retrieval to maximize the sensitivity of the search strategy, the included population predominantly consisted of squamous cell carcinoma cases. The current meta‐analysis did not include case reports or research on preclinical models.

In our primary search, we looked for articles published in the Latin alphabet, regardless of the language used, using the Medline (1966–2024), Scopus (2004–2024), Clinicaltrials.gov (2008–2024), Cochrane Central Register of Controlled Trials CENTRAL (1999–2024), and Google Scholar (2004–2024) databases in addition to the reference lists of electronically retrieved full text papers. Before the search began, the decision was made to use internet translation tools to translate languages other than English, French, German, Italian, and Spanish. Our most recent search was scheduled for June 30, 2024. Figure 1 provides a quick overview of our search approach, which includes the text phrases “vulvar cancer,” “lymphovascular space,” “LVSI,” and “survival.” The complete reproducible search strategy for each database, including Boolean operators, MeSH terms, and database‐specific syntax, is provided in Data S1.

**FIGURE 1:**
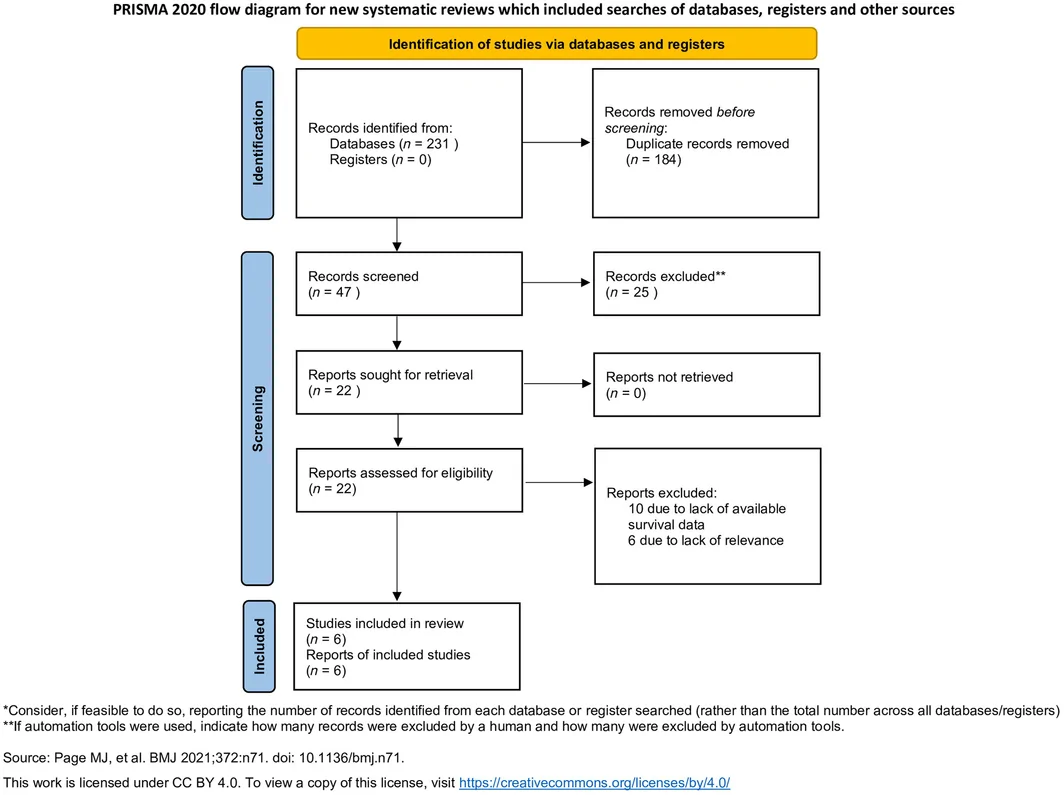
Search plot strategy.

### 2.3 Study selection

Three steps were taken to retrieve the relevant studies. First, the Rayyan program was used to deduplicate the articles that were retrieved. Two authors (MP and MF) then manually screened the titles and abstracts of all remaining electronic papers to determine their eligibility. Studies deemed possibly eligible were chosen for full‐text assessment in the last stage of the research selection procedure. Any disagreements that surfaced beyond this point were settled by agreement among all authors.

### 2.4 Data extraction

During the current systematic review's design, outcome measures were predetermined. A modified data form based on Cochrane's data collection form for intervention reviews for randomized controlled trials (RCTs) and non‐RCTs was used to extract data. Assessing the variations in survival rates (overall survival, or OS, and progression free survival, or PFS) between vulvar cancer patients with and without LVSI was the main goal of our investigation. Assessment of inguinofemoral metastases was considered an exploratory secondary objective whenever extractable subgroup data according to LVSI status were available. To reduce the risk of underestimating disease recurrence and survival outcomes due to insufficient follow‐up duration, a minimum median follow‐up period of 3 years was established as an eligibility criterion for study inclusion. In addition to variations in other histological criteria, such as tumor stage, presence of positive surgical margins, tumor grade, and tumor size, between patients with and without LVSI, differences in crude time intervals until disease relapse and mortality were also taken into consideration as secondary outcomes.

### 2.5 Assessment of risk of bias

Two authors (MF and VP) evaluated the methodological quality of the included observational studies using the Newcastle–Ottawa scale score, which evaluates the risk of bias in observational studies by evaluating the study groups' selection (maximum rating 4 points), their comparability (maximum rating 2 points: 1 for comparable histological subtypes and another for the actual stage of the disease at pathology analysis), and the outcome of interest (which was predefined as a minimum 3‐year median follow‐up as indicated by the authors or by a gap of at least 3 years between the last patient recruitment and the article's publication) (maximum rating 3 points).^17^ The certainty of evidence for each pooled outcome was additionally assessed using the GRADE (Grading of Recommendations Assessment, Development and Evaluation) framework, considering risk of bias, inconsistency, indirectness, imprecision, and publication bias (Data S1).

### 2.6 Data synthesis

The meta function in RStudio was used to conduct a statistical meta‐analysis (RStudio Team, 2015; http://www.rstudio.com/ RStudio: Integrated Development for R. RStudio, Inc., Boston, MA). Since the significant methodological heterogeneity of observational studies precludes the assumption of comparable effect sizes among studies included in meta‐analyses, statistical heterogeneity was not taken into account when evaluating the appropriate model (fixed effects or random effects) of statistical analysis.^18^ Multivariate models were favored over aggregated data from univariate analyses to decrease the impact of potential confounders, such as disease stage, differentiation grade, and other factors, such as lymphovascular space involvement and tumor molecular profile. This study used 95% CIs. Instead of using the conventional Dersimonian–Laird random effects model analysis (REM), we used the Hartung–Knapp–Sidik–Jonkman to compute the pooled hazard ratio (HR) of survival and 95% confidence intervals (CI). We chose to employ this model because recent studies show that it is more effective than the Dersimonian–Laird model at explaining the heterogeneity of the included observational studies, which are likely to have quite different methodologies.^19^ Odds ratios for 5‐year survival outcomes were calculated using directly reported survival proportions from included studies whenever available and were not reconstructed from Kaplan–Meier survival curves.

We took into account the Egger's test as a statistical technique to assess the potential for publication bias when designing this systematic review. The intervention effect estimates and their standard errors, which are weighted by their inverse variance, are considered in this method's linear regression analysis.^20^ It is only deemed significant when there is strong evidence, and in order to guarantee the proper trustworthiness of the results returned, a minimum cut‐off of 10 studies was established for each analyzed outcome.^21^

We used Rücker's limit meta‐analysis, which allows the examination of small study effects in the meta‐analytic pooled effect, to investigate the possible impact of aggregate results from smaller research. *p*‐curve analysis was also used to rule out the possibility of data tampering (*p*‐hacking) and examine the veracity of the combined findings of the included research.

### 2.7 Prediction intervals

To assess the estimated influence that is anticipated to be observed by subsequent research in the field, prediction intervals were also computed using RStudio's meta function. Prediction interval estimation expresses the current heterogeneity at the same scale as the outcome under research and takes into account the diversity of outcomes across studies.

## 3 RESULTS

Overall, 22 articles were selected in full text and screened.^13^, ^14^, ^15^, ^22^, ^23^, ^24^, ^25^, ^26^, ^27^, ^28^, ^29^, ^30^, ^31^, ^32^, ^33^, ^34^, ^35^, ^36^, ^37^, ^38^, ^39^ Of those, six articles were selected for inclusion,^15^, ^22^, ^23^, ^24^, ^25^, ^27^ as the remainder were either not relevant to the subject or did not provide survival outcomes, which were a prerequisite for inclusion in the present systematic review. Methodologically significant discrepancies were observed among included studies, which included populations that referred to a wide timespan (Table 1), an observation that renders clear the fact that patients might have received heterogeneous treatments. This was pointed out in one of the studies,^25^ whereas the remaining four did not provide evidence on the actual differentiation of treatment plans over the years that were reviewed. Most of the studies included as a single prerequisite for inclusion of patients the presence of squamous cancer on the histology report, whereas two studies differentiated. The first one included extended criteria as a pre‐requisite for inclusion of patients,^23^ whereas the second study included patients with non‐squamous histology as well (one case with leiomyosarcoma, one case with verrucous carcinoma, and one case with a malignant peripheral nerve sheath tumor).^22^ To evaluate the impact of the potential differences of these two studies, we added leave‐one‐out analysis in our sensitivity analysis. None of the studies that were included in this systematic review subgrouped patients according to their LVSI status (results were provided from multivariate survival analyses); hence, in terms of patient selection, significant bias could obscure the findings of this meta‐analysis (Table 2). The certainty of evidence was considered very low for progression‐free survival and low for overall survival (Data S1). This resulted in significant discrepancies in terms of patient characteristics, including tumor size, tumor grade, type of surgery, rates of lymphadenectomy, and presence of disease on surgical margins of specimens, as denoted in Table 3.

**TABLE 1:** Methodological characteristics of included studies.

Study, Year	Study	Country – years	Patients	Inclusion criteria	Exclusion criteria
Raspagliesi, 2006	Retrospective	Italy 1978–1995	389	Patients that underwent radical excision for squamous vulvar carcinoma with a tumor extension of at least 1 mm depth of invasion	Patients with other histological types and those who underwent neoadjuvant chemo‐radiotherapy or had less than 1 mm in depth of invasion
Ayhan, 2008	Retrospective	Turkey 1982–2004	91	Patients that underwent radical excision for squamous vulvar carcinoma	Histological type other than non‐squamous vulvar carcinoma
Chen, 2009	Retrospective	China 1980–2002	100	Patients that underwent radical excision for squamous vulvar carcinoma	Histological type other than non‐squamous vulvar carcinoma
Aragona, 2014	Retrospective	Argentina 1992–2012	194	Patients that underwent radical excision for squamous vulvar carcinoma with depth of stromal invasion >1 mm, tumor size >2 cm, and tumor‐free margin of at least 8 mm (after formalin fixation)	Patients who underwent neoadjuvant therapy due to locoregionally advanced vulvar cancer, patients with an indication for adjuvant therapy who have not completed such treatment according to the technique described below, patients with distant metastasis (stage IVB), and patients with concomitant malignancies at the time of diagnosis
Alanyali, 2016	Retrospective	Turkey 2000–2011	44	Patients with vulvar cancer treated with postoperative or definitive RT/RCT	N/A
Muangchang, 2021	Retrospective	China 2006–2017	125	Patients that underwent radical excision for squamous vulvar carcinoma	Histological type other than non‐squamous vulvar carcinoma

Abbreviations: RCT, chemoradiotherapy; RT, radiotherapy.

**TABLE 2:** Assessment of the methodological quality of included studies.

Study	Selection				Comparability	Outcome		
	Adequacy of case definition	Representativeness of cases	Selection of controls	Definition of controls	Comparability (Grade/Stage)	Assessment of outcome	Adequate follow‐up period	Adequate follow‐up
Raspagliesi, 2006	√	‐	‐	‐	–^b^	√	√	√
Ayhan, 2008	√	‐	‐	‐	–^a^	√	√	√
Chen, 2009	√	‐	‐	‐	–^b^	√	√	√
Aragona, 2014	√	‐	‐	‐	–^b^	√	√	√
Alanyali, 2016	√	‐	‐	‐	–^a^	√	√	‐
Muangchang, 2021	√	‐	‐	‐	–^a^	√	√	√

Abbreviation: LVSI, lymphovascular space involvement.

^a^ Univariable Kaplan–Meier analysis was used to evaluate the impact of LVSI on survival outcomes.

^b^ Multivariable analysis with the Cox regression model accounting for factors that significantly differed among patients with LVSI and those without LVSI was used to evaluate the impact of LVSI on survival outcomes.

**TABLE 3:** Differences in tumor and treatment characteristics among included studies. Differences among LVSI positive and LVSI negative patients were not available among included studies.

Study, Year	Median age (years)	Tumor size (mm)	Tumor grade	Type of vulvar surgery	Depth of stromal invasion (mm)	Lymphadenectomy (Yes/No)	Stage	Histopathology	Surgical margin (negative/positive)
Raspagliesi, 2006	64.4 (28–86)	<20: 86 (27, 1%) 20–40: 119 (37, 6%) >40: 44 (13, 8%)	N/A	N/A	N/A	N/A	N/A	N/A	1–2: 71 (18, 2%) >2: 39 (10%)
Chen, 2009	<40: 5 (5%) ≥40–50: 12 (12%) ≥50–60: 15 (15%) ≥60–70: 38 (38%) ≥70: 30 (30%)	≤20: 14 (16, 9%) 20–40: 24 (28, 9%) ≥40: 45 (54, 2%) Missing: 17	1: 46 (46%) 2: 45 (45%) 3: 9 (9%)	Radical total vulvectomy: 62 (62%) Radical partial vulvectomy: 2 (2%) Extended radical vulvectomy: 33 (33%) Local excision: 3 (3%)	N/A	Yes: 80 (80%) No: 20 (20%)	I–II: 28 (35%) III: 38 (47, 5%) IV: 14 (17, 5%) N/A: 20	Squamous: 100 (100%)	Negative: 92 (92%) Positive: 8 (8%)
Ayhan, 2008	62 ± 14	<10: 22 (24, 1%) ≥10: 69 (75, 8%)	1: 67 (73, 6%) 2: 17 (18, 6%) 3:7 (7, 6%)	Radical total vulvectomy: 79 (86, 8%) Local excision: 12 (13, 1)	<1: 60 (65, 9%) ≥1: 31 (34%)	Yes: 34 (37, 3%) No: 57 (62, 6%)	I: 14 (15, 3%) II: 42 (46, 1%) III: 26 (28, 5%) IV: 9 (9, 8%)	Squamous: 91 (100%)	Negative: 86 (94, 5) Positive: 5 (5, 4%)
Aragona, 2014	<44: 11 (5, 7%) 45–64: 68 (35%) 65–70: 37 (19%) >70: 78 (40, 2%)	>20–39, 9: 85 (43,8) 40–59, 9: 54 (27, 8%) 60–79, 9: 33 (17%) ≥80: 22 (11, 3%)	1: 66 (34%) 2: 96 (49, 5%) 3: 32 (16, 5%)	Radical vulvectomy: 139 (71, 6%) Wide excision:50 (25, 8%) Pelvic exenteration: 5 (2, 6%)	>1–4: 110 (56, 7%) >4: 84 (43, 3%)	Yes: 194 (100%)	IB: 73 (37, 8%) II: 25 (12, 8%) III: 92 (47, 4%) IVA: 4 (2%)	N/A	N/A
Alanyali, 2016	68	N/A	N/A	Radical vulvectomy: 8 (18, 2%) Simple vulvectomy: 16 (36, 4%) Wide local excision: 6 (13, 6%)	N/A	Yes: 16 (36, 3%) No: 28 (63, 6%)	IB: 11 (25%) II: 10 (22, 7%) III: 11 (25%) IVA: 12 (27, 3%)	Squamous: 41(93,2%) Leiomyosarcoma: 1 (2, 3%) Verrucous: 1 (2, 3%) Malign peripheral nerve sheet tumor: 1(2, 3%)	Negative: 12 (27, 3%) Positive: 5 (16, 7%) Close (≤8 mm): 13 (43, 3%)
Muangchang, 2021	N/A	≤25: 47 (37, 6%) >25: 58 (46, 4%) N/A: 20 (16%)	1: 90 (72%) 2 + 3: 32 (25, 6%) N/A: 3 (2, 4%)	N/A	N/A	N/A	I: 48 (38, 4%) II: 31 (24, 8%) III: 37 (29, 6%) IV: 9 (7, 2%)	Squamous: 125 (100%)	Negative: 64 (51, 2%) Positive: 49 (39, 2%) N/A: 12 (9, 6%)

*Note*: N/A, data were not available in this study.

### 3.1 Primary variables

Significant differences were noted in the PFS of patients with LVSI compared to that of patients without LVSI (HR 2.79, 95% CI 1.27, 6.14, Figure 2). Statistical heterogeneity of included studies was significant (*I* ^2^‐test = 68%), and the results of the sensitivity analysis using the fixed effects model that assumes no true heterogeneity among studies revealed a similar summary effect estimate compared to that of the random effects analysis (HR 2.38, 95% CI 1.68, 3.38). Prediction intervals were not statistically significant, indicating that future studies might result in non‐significant results. None of the included studies identified as a statistical outlier in the PFS analysis, and the study by Aragona et al.^23^ did not significantly affect the statistical significance of the primary findings of this systematic review (refer to Appendix S1). The analysis for small‐study effects did not reveal significant results (*P* = 0.251). Analysis of p‐hacking revealed that the summary effect estimate provided an evidential and potentially unbiased value. Consequently, the 5‐year PFS was considerably lower among patients without LVSI (OR 0.34, 95% CI 0.12, 0.96, Figure 2), and the sensitivity analysis revealed similar summary effect estimate in the fixed effects analysis (OR 0.33, 95% CI 0.16, 0.71) with minimal statistical heterogeneity (*I* ^2^‐test = 0%).

**FIGURE 2:**
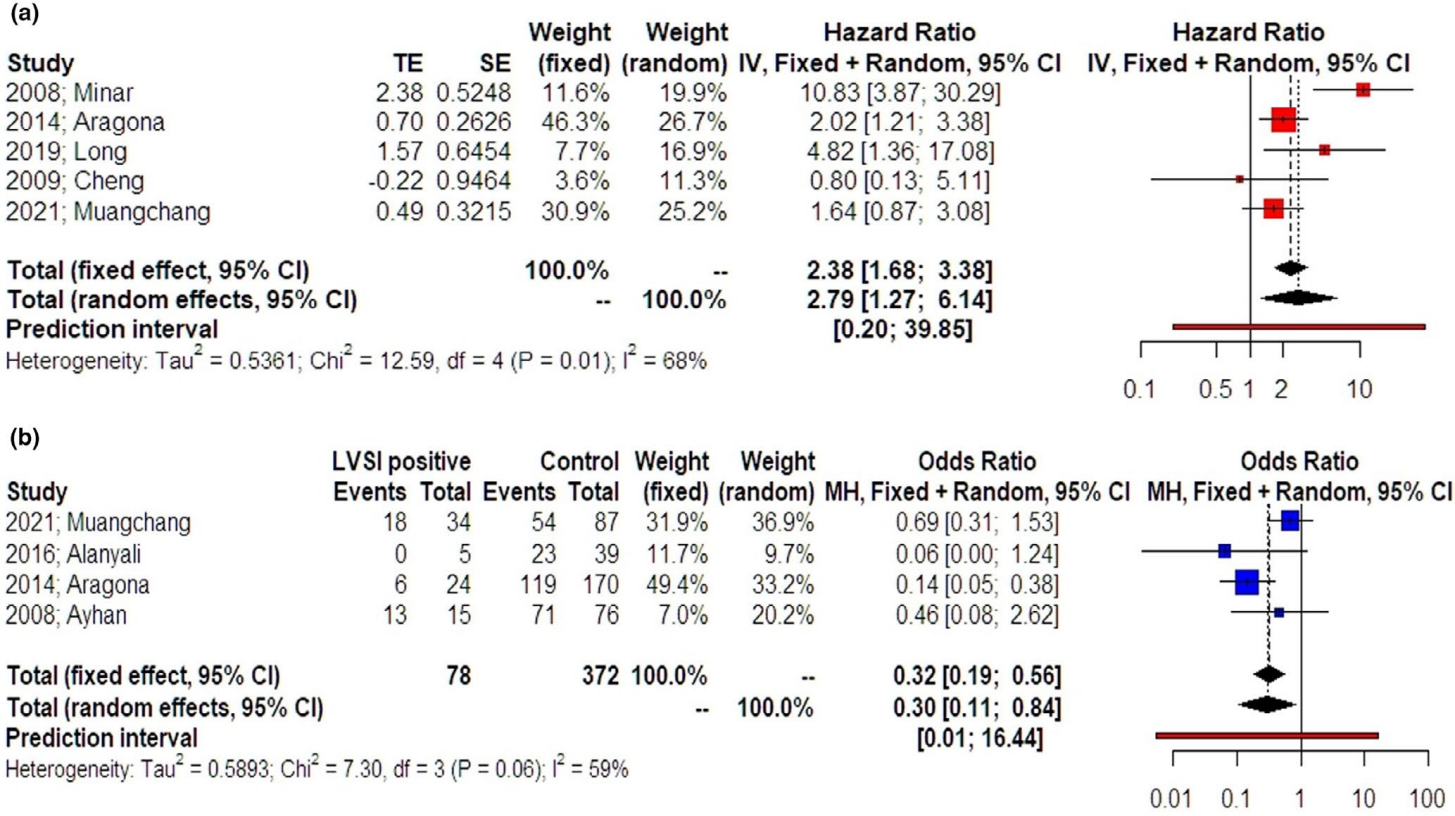
(a) Hazard ratio of progression free survival (upper forest plot) and (b) odds ratios of 5‐year disease free survival (lower forest plot). Forest plot analysis: Vertical line, “no difference” point between the two groups. Red squares, hazard ratios of disease‐free survival of included studies; blue squares, odds ratios of 5‐year disease‐free survival of included studies; horizontal black lines, 95% CI of included studies; diamond, pooled hazard and odds ratios retrieved from the outcomes of the meta‐analysis and 95% CI for all studies; horizontal red line, prediction intervals. The weight of included studies is depicted for fixed and random effects model separately. Considerable statistical heterogeneity was observed for progression‐free survival (*I* ^2^ = 68%), whereas no substantial heterogeneity was noted for 5‐year disease‐free survival (*I* ^2^ = 0%).

Similar to the PFS, the OS of patients with LVSI was substantially lower compared to that of patients without LVSI (HR 1.66, 95% CI 1.18, 2.35, Figure 3). Statistical heterogeneity was absent in this analysis (*I* ^2^‐test = 0%), and the sensitivity analysis using the fixed effects model revealed a similar effect estimated. Prediction intervals denoted that the results of future studies might considerably deviate from those of the present analysis. None of the included studies identified as an outlier in the random effects model, and leave‐one‐out analysis revealed that the study of Aragona et al. did not significantly alter the results of our study.^3^ Analysis for small study effects did not reveal significant results (*P* = 0.242). *p*‐curve analysis was not possible due to the small number of retrieved articles with statistically significant differences among LVSI positive and LVSI negative patients. The 5‐year specific OS of patients without LVSI was similarly considerably smaller (OR 0.30, 95% CI, 0.11, 0.84). Differences in the summary effect estimate between the random effect and fixed effect model were minimal, despite the considerable statistical heterogeneity that was observed (*I* ^2^‐test = 59%).

**FIGURE 3:**
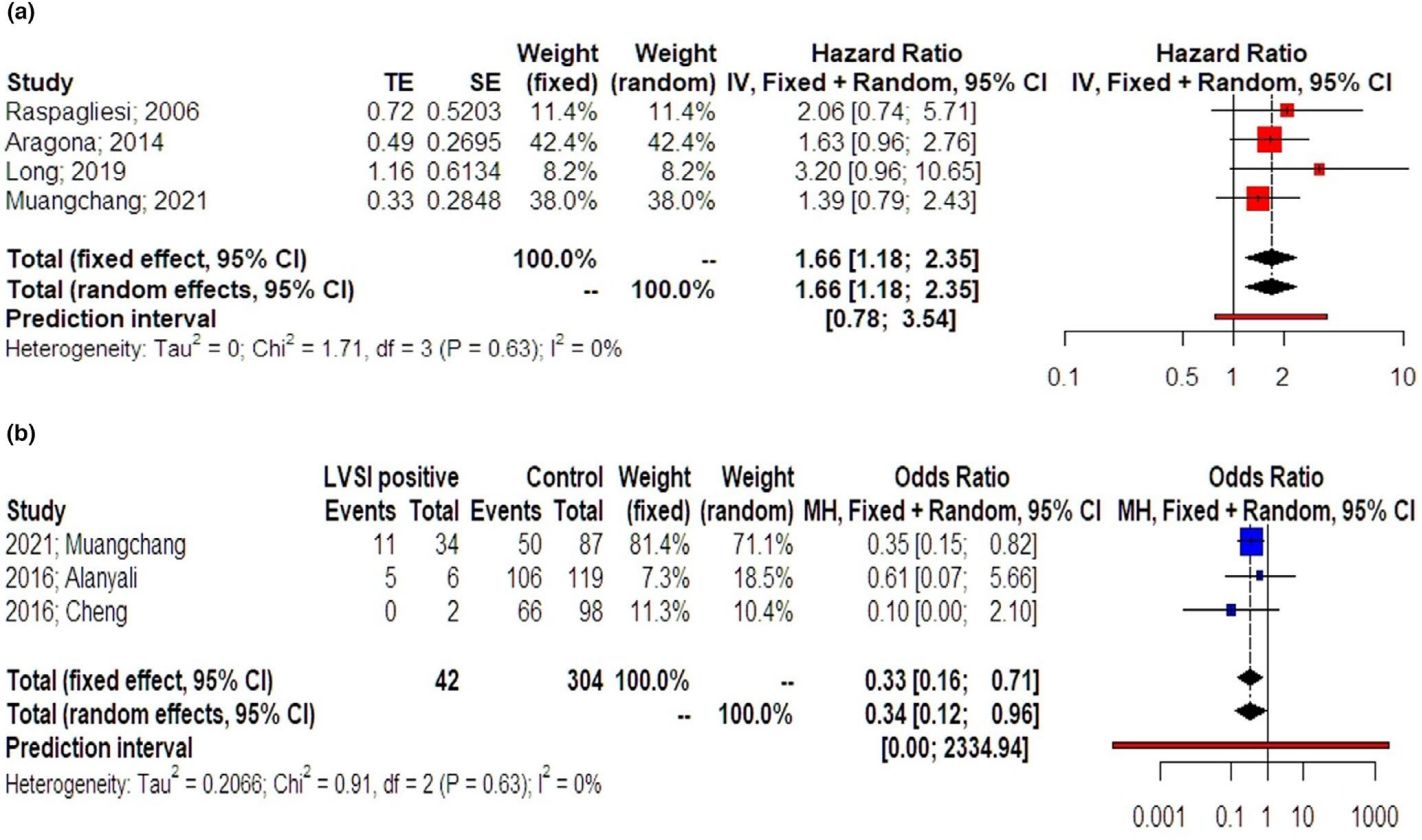
(a) Hazard ratio of overall survival (upper forest plot) and (b) odds ratios of 5‐year overall survival (lower forest plot). Forest plot analysis: Vertical line, “no difference” point between the two groups; red squares, hazard ratios of overall survival of included studies; blue squares, odds ratios of 5‐year overall survival of included studies; horizontal black lines, 95% CI of included studies; diamond, pooled hazard and odds ratios retrieved from the outcomes of the meta‐analysis and 95% CI for all studies; horizontal red line, prediction intervals. The weight of included studies is depicted for fixed and random effects model separately. Minimal statistical heterogeneity was observed for overall survival (*I* ^2^ = 0%), whereas moderate heterogeneity was noted for 5‐year overall survival (*I* ^2^ = 59%).

### 3.2 Secondary variables

During inception of the present systematic review, we opted to evaluate differences in tumor stage, the percentage of patients' positive surgical margins, tumor grade, and tumor size among patients with LVSI and those without LVSI as well as to evaluate the presence of groin metastases. However, none of the included studies provided relevant data, as LVSI was considered as a secondary variable of vulvar cancer survival for which subgroup analysis was not available.

## 4 DISCUSSION

### 4.1 Principal findings

The findings of our systematic review suggest that lymphovascular space involvement is associated with worse survival outcomes in vulvar cancer. These findings seem to be independent of the sample size that the selected studies used, as the actual impact of small study effects was minimal. It is important to also note that there were no significant outliers that could potentially affect the results of the overall effect estimate, therefore increasing the validity of our findings. Significant heterogeneity was, however, noted among included studies in terms of patient selection and characteristics of included patients, which could result in significant bias. This is also reflected by the wide prediction intervals in both PFS and OS outcomes, which denote the possibility of significant differences in future studies. Therefore, the conclusions of the present meta‐analysis should primarily be interpreted within the context of vulvar squamous cell carcinoma.

### 4.2 Evidence from the literature

Lymphovascular space involvement is defined as the presence of tumor cells in endothelial‐lined vascular spaces^40^ and considered a precursor of lymphatic and, potentially, hematogenous metastases in various gynecological cancers. In 2023, the International Federation of Obstetrics and Gynecology (FIGO) introduced LVSI as a factor that directly affects the staging of non‐aggressive histological types, therefore denoting its importance as a single factor that merits consideration during planning of adjuvant treatment.^41^ More than 2 decades ago, Sedlis introduced LVSI as one of the three most important factors, along with depth of invasion and tumor size, affecting the decision to proceed in adjuvant pelvic radiation for patients with early stage disease offered radical hysterectomy and pelvic lymphadenectomy.^42^ Considering the decisive role of LVSI in these tumors, it would be prudent to support its importance in patients with vulvar cancer. However, to date, none of the international societies that have issued practice guidelines in this topic supports a different approach of vulvar cancer patients with LVSI.^5^, ^9^, ^43^, ^44^ This reflects a significant gap in current understanding of the actual value of LVSI, which is, nevertheless, reported as a negative prognostic factor by other researchers as well, despite the fact that their studies did not provide the appropriate amount of data to be included in this systematic review.^13^, ^28^, ^29^, ^30^

### 4.3 Limitations of this study

Several limitations of the present meta‐analysis should be acknowledged. First, all included studies were retrospective observational cohorts, introducing an inherent risk of selection bias, information bias, and residual confounding. Moreover, LVSI was not consistently evaluated as a primary prognostic variable but rather as an adjunct histopathological factor. To minimize this limitation, we preferentially incorporated findings derived from multivariable‐adjusted analyses whenever these were available. Second, considerable methodological and clinical heterogeneity existed among the included studies. The retrieved cohorts spanned several decades and differed substantially regarding patient populations, disease stage distribution, histological composition, surgical management, lymphadenectomy rates, adjuvant treatment strategies, follow‐up duration, and statistical adjustment methods. These differences likely contributed to the observed heterogeneity of pooled estimates and limit the generalizability of the findings, particularly among patients with early‐stage disease who might potentially benefit most from prognostic stratification based on LVSI status.

Another important limitation is the relatively small number of eligible studies and patients included in the quantitative synthesis. However, vulvar cancer remains a rare gynecologic malignancy, and studies specifically evaluating the prognostic impact of LVSI on survival outcomes are limited. Consequently, subgroup analyses according to tumor stage or histological subtype, as well as meaningful meta‐regression analyses, were not feasible. In addition, the modest pooled sample size and limited number of studies with long‐term follow‐up might have reduced the statistical power of certain analyses and restricted the ability to reliably assess publication bias. Nevertheless, despite these limitations, the present study provides the most comprehensive synthesis of currently available evidence regarding the prognostic role of LVSI in vulvar cancer. Therefore, although the pooled findings suggest that LVSI is associated with worse survival outcomes, the results should be interpreted cautiously until larger prospective multicenter studies provide higher‐quality evidence.

From a clinical perspective, the findings of the present meta‐analysis suggest that LVSI might represent an additional prognostic histopathological factor in vulvar cancer patients. The presence of LVSI likely reflects a biologically more aggressive tumor phenotype with increased metastatic potential and might partially explain the worse survival outcomes observed among affected patients. Although current treatment algorithms are primarily guided by tumor stage, nodal involvement, and surgical margin status, prognostic stratification in vulvar cancer remains relatively limited, particularly among patients with early‐stage disease who might still experience recurrence despite otherwise favorable clinicopathological characteristics. In this context, routine assessment and reporting of LVSI status might provide additional prognostic information that could help identify higher‐risk patient subgroups requiring closer surveillance or more individualized therapeutic approaches.

Nevertheless, current evidence remains insufficient to directly modify adjuvant treatment recommendations, as available data are largely retrospective and heterogeneous. Future prospective multicenter studies are warranted to determine whether LVSI could be integrated into validated prognostic models and eventually support individualized decisions regarding adjuvant treatment selection, surveillance intensity, and long‐term follow‐up strategies.

## 5 CONCLUSION

The findings of the present meta‐analysis suggest that lymphovascular space involvement should be considered a significant predictor of disease recurrence and overall survival in patients suffering from squamous cell vulvar carcinoma. It should be noted, however, that this information is limited by the methodological heterogeneity of included studies and the heterogeneity of included patients, which can result in significant bias, despite the fact that we opted to include data from multivariate analyses when these were available. Considering the actual impact of LVSI on decision‐making processes in other gynecological cancers, it seems reasonable to support the conduct of further research in vulvar cancer patients as well. This should ideally target patients with early‐stage disease to evaluate if LVSI alone suffices to support upstaging of patients and their selection for adjuvant therapy as well.

## AUTHOR CONTRIBUTIONS

Concept: Vasilios Pergialiotis, Dimitrios Haidopoulos. Design: Vasilios Pergialiotis, Maria Fanaki, Dimitrios Haidopoulos. Data Collection or Processing: Michail Panagiotopoulos, Maria Fanaki. Analysis or Interpretation: Vasilios Pergialiotis, Vasilios Lygizos, Dimitrios Efthimios Vlachos. Literature search: Michail Panagiotopoulos, Maria Fanaki, Nikolaos Thomakos. Writing: All authors.

## FUNDING INFORMATION

The authors declare that no funds, grants, or other support were received during the preparation of this manuscript.

## CONFLICT OF INTEREST STATEMENT

The authors have no relevant financial or non‐financial interests to disclose.

## CONSENT

The review is based on aggregated data that have been already published in international literature. Patient consent and institutional review board approval were, therefore, waived.

## PROSPERO REGISTRATION NUMBER

CRD42024545939.

## Supporting information


Appendix S1.

Data S1.

Table S1.

## Data Availability

Data sharing is not applicable to this article as no new data were created or analyzed in this study.
