# Supplementary material for: Lymphovascular space involvement as a prognostic factor for survival outcomes in vulvar cancer: A systematic review and meta‐analysis
Source: Int J Gynaecol Obstet. 2026 Jun 29;175(3):1188–98. doi: 10.1002/ijgo.71157 (PMC13769344; doi:10.1002/ijgo.71157)
Supplement: Supplementary file 1 [file IJGO-175-1188-s001.docx]

| **Outcome** | **Number of Studies** | **Study Design** | **Risk of Bias** | **Inconsistency** | **Indirectness** | **Imprecision** | **Publication Bias** | **Overall Certainty of Evidence (GRADE)** |
| --- | --- | --- | --- | --- | --- | --- | --- | --- |
| Progression-free survival (PFS) | 5 studies | Retrospective observational cohort studies | Serious | Serious | Not serious | Serious | Undetected* | **Very low** |
| Overall survival (OS) | 4 studies | Retrospective observational cohort studies | Serious | Not serious | Not serious | Serious | Undetected* | **Low** |

**Table S1. GRADE assessment of certainty of evidence for progression-free survival (PFS) and overall survival (OS) outcomes in vulvar cancer patients according to lymphovascular space involvement (LVSI) status. GRADE: Grading of Recommendations Assessment, Development and Evaluation.**

The certainty of evidence for progression-free survival was judged as very low according to the GRADE framework. The evidence was downgraded due to the retrospective observational design of included studies and important methodological limitations, substantial clinical and statistical heterogeneity among studies (I²=68%), and imprecision related to small sample sizes and wide confidence/prediction intervals.

The certainty of evidence for overall survival was considered low. Although all included studies were retrospective observational cohorts with inherent risk of bias, statistical heterogeneity for overall survival was low (I²=0%), supporting greater consistency of findings across studies. Nevertheless, the evidence was downgraded due to methodological limitations and imprecision associated with limited sample size and wide prediction intervals.
