# Supplementary material for: Lymphovascular space involvement as a prognostic factor for survival outcomes in vulvar cancer: A systematic review and meta‐analysis
Source: Int J Gynaecol Obstet. 2026 Jun 29;175(3):1188–98. doi: 10.1002/ijgo.71157 (PMC13769344; doi:10.1002/ijgo.71157)
Supplement: Supplementary file 2 [file IJGO-175-1188-s002.docx]

**Data S1.** Complete electronic search strategy

The electronic literature search was conducted using Medline (via PubMed), Scopus, Cochrane Central Register of Controlled Trials (CENTRAL), ClinicalTrials.gov, and Google Scholar from database inception until June 30, 2024.

The following search strategies were used:

PubMed (Medline)

("vulvar cancer"[Title/Abstract] OR "vulval cancer"[Title/Abstract] OR "vulvar neoplasm*"[Title/Abstract] OR "vulvar carcinoma"[Title/Abstract])
AND
("lymphovascular space invasion"[Title/Abstract] OR "lymphovascular invasion"[Title/Abstract] OR LVSI[Title/Abstract])
AND
(survival[Title/Abstract] OR prognosis[Title/Abstract] OR recurrence[Title/Abstract] OR metastasis[Title/Abstract])

Scopus

TITLE-ABS ("vulvar cancer" OR "vulval cancer" OR "vulvar neoplasm*" OR "vulvar carcinoma")
AND
TITLE-ABS ("lymphovascular space invasion" OR "lymphovascular invasion" OR LVSI)
AND
TITLE-ABS (survival OR prognosis OR recurrence OR metastasis)

Cochrane Central Register of Controlled Trials (CENTRAL)

("vulvar cancer" OR "vulval cancer" OR "vulvar carcinoma")
AND
("lymphovascular space invasion" OR "lymphovascular invasion" OR LVSI)
AND
(survival OR prognosis OR recurrence OR metastasis)

ClinicalTrials.gov

Condition or disease:
“Vulvar cancer”

Other terms:
“lymphovascular space invasion” OR LVSI

Google Scholar

("vulvar cancer" OR "vulval cancer")
AND
("lymphovascular space invasion" OR LVSI)
AND
(survival OR prognosis)

Reference lists of all eligible full-text articles were additionally screened manually to identify potentially relevant studies.

No language restrictions were applied for studies published in the Latin alphabet.
