# Supplementary material for: Lymphovascular space involvement as a prognostic factor for survival outcomes in vulvar cancer: A systematic review and meta‐analysis
Source: Int J Gynaecol Obstet. 2026 Jun 29;175(3):1188–98. doi: 10.1002/ijgo.71157 (PMC13769344; doi:10.1002/ijgo.71157)
Supplement: Supplementary file 3 [file IJGO-175-1188-s003.docx]

# Appendix S1

## Overall survival

```
Small study effects analysis

                        HR            95%-CI        HR           95%-CI
Raspagliesi; 2006   2.0600 [0.7430;  5.7114]    0.9161 [0.3304; 2.5399]
Aragona; 2014       1.6300 [0.9612;  2.7644]    0.9161 [0.5402; 1.5536]
Long; 2019          3.2002 [0.9617; 10.6487]    0.9161 [0.2753; 3.0483]
Muangchang; 2021    1.3890 [0.7949;  2.4273]    0.9161 [0.5242; 1.6009]

Result of limit meta-analysis:

 Random effects model     HR           95%-CI     z   pval
    Adjusted estimate 0.9161 [0.3179; 2.6401] -0.16 0.8711
  Unadjusted estimate 1.6648 [1.1801; 2.3485]  2.90 0.0037

Quantifying heterogeneity:
tau^2 = 0; I^2 = 0.0% [0.0%; 84.7%]; G^2 = 0.0%

Test of heterogeneity:
    Q d.f. p-value
 1.71    3  0.6340

Test of small-study effects:
 Q-Q' d.f. p-value
 1.37    1  0.2421

Test of residual heterogeneity beyond small-study effects:
   Q' d.f. p-value
 0.34    2  0.8416

Influential analysis (Fixed effect model)

                                 HR           95%-CI p-value   tau^2  I^2
Omitting Raspagliesi; 2006   1.6199 [1.1239; 2.3346]  0.0097  0.0000  0.0%
Omitting Aragona; 2014       1.6909 [1.0744; 2.6611]  0.0232  0.0000  0.0%
Omitting Long; 2019          1.5705 [1.0967; 2.2490]  0.0137  0.0000  0.0%
Omitting Muangchang; 2021    1.8602 [1.2017; 2.8796]  0.0054  0.0000  0.0%

Pooled estimate              1.6648 [1.1801; 2.3485]  0.0037  0.0000  0.0%


Influential analysis (Random effects model)

                                 HR           95%-CI p-value   tau^2  I^2
Omitting Raspagliesi; 2006   1.6199 [1.1239; 2.3347]  0.0097  0.0000  0.0%
Omitting Aragona; 2014       1.6909 [1.0744; 2.6611]  0.0232  0.0000  0.0%
Omitting Long; 2019          1.5705 [1.0967; 2.2490]  0.0137  0.0000  0.0%
Omitting Muangchang; 2021    1.8602 [1.2017; 2.8796]  0.0054  0.0000  0.0%

Pooled estimate              1.6648 [1.1801; 2.3485]  0.0037  0.0000  0.0%
```

## Progression free survival

```
Small study effects analysis

                       HR            95%-CI        HR            95%-CI
2008; Minar        10.8298 [3.8718; 30.2922]    9.3036 [3.3261; 26.0232]
2014; Aragona       2.0230 [1.2091;  3.3848]    2.1286 [1.2722;  3.5614]
2019; Long          4.8201 [1.3605; 17.0775]    4.8179 [1.3599; 17.0696]
2009; Cheng         0.7990 [0.1250;  5.1065]    1.6038 [0.2509; 10.2501]
2021; Muangchang    1.6390 [0.8728;  3.0778]    1.7949 [0.9558;  3.3706]

Result of limit meta-analysis:

 Random effects model     HR           95%-CI    z   pval
    Adjusted estimate 3.0744 [1.3011; 7.2646] 2.56 0.0105
  Unadjusted estimate 2.7939 [1.2714; 6.1397] 2.56 0.0105

Quantifying heterogeneity:
tau^2 = 0.5361; I^2 = 68.2% [18.1%; 87.7%]; G^2 = 91.3%

Test of heterogeneity:
     Q d.f. p-value
 12.59    4  0.0135

Test of small-study effects:
  Q-Q' d.f. p-value
  1.31    1  0.2516

Test of residual heterogeneity beyond small-study effects:
    Q' d.f. p-value
 11.28    3  0.0103

Influential analysis (Fixed effect model)

                                 HR           95%-CI p-value   tau^2  I^2
Omitting Raspagliesi; 2006   1.6199 [1.1239; 2.3346]  0.0097  0.0000  0.0%
Omitting Aragona; 2014       1.6909 [1.0744; 2.6611]  0.0232  0.0000  0.0%
Omitting Long; 2019          1.5705 [1.0967; 2.2490]  0.0137  0.0000  0.0%
Omitting Muangchang; 2021    1.8602 [1.2017; 2.8796]  0.0054  0.0000  0.0%

Pooled estimate              1.6648 [1.1801; 2.3485]  0.0037  0.0000  0.0%

Influential analysis (Random effects model)

                                 HR           95%-CI p-value   tau^2  I^2
Omitting Raspagliesi; 2006   1.6199 [1.1239; 2.3347]  0.0097  0.0000  0.0%
Omitting Aragona; 2014       1.6909 [1.0744; 2.6611]  0.0232  0.0000  0.0%
Omitting Long; 2019          1.5705 [1.0967; 2.2490]  0.0137  0.0000  0.0%
Omitting Muangchang; 2021    1.8602 [1.2017; 2.8796]  0.0054  0.0000  0.0%

Pooled estimate              1.6648 [1.1801; 2.3485]  0.0037  0.0000  0.0%


P-curve analysis
 -----------------------
- Total number of provided studies: k = 5
- Total number of p<0.05 studies included into the analysis: k = 3 (60%)
- Total number of studies with p<0.025: k = 3 (60%)

Results
 -----------------------
                    pBinomial  zFull pFull  zHalf pHalf
Right-skewness test     0.125 -3.047 0.001 -2.207 0.014
Flatness test           1.000  1.657 0.951  2.602 0.995
Note: p-values of 0 or 1 correspond to p<0.001 and p>0.999, respectively.
Power Estimate: 83% (33.8%-98.2%)

Evidential value
 -----------------------
- Evidential value present: yes
- Evidential value absent/inadequate: no
```
